# Our New York Correspondence

**Published:** 1859-09

**Authors:** 


### OUR NEW YORK CORRESPONDENCE.

We desire to direct attention to a second interesting letter from our New York correspondent. In the conclusion will be found a defence of himself against the charge of unfairness (in his first letter,) preferred by the Editor of the *Philadelphia Medical Reporter*, who thinks Dr. O'Keefe should have remained in Philadelphia to have been treated for an affection of the eyes.

We are not disposed to enter the controversy, as we have not been to the North for a number of years, and cannot, therefore, speak understandingly of the relative advantages of New York and Philadelphia, so far as the facilities for acquiring Medical knowledge are concerned, and especially as we expect, in a few weeks, to judge for ourselves, and to give our readers the benefit of our observations.

